# Efficacy and Safety of Chlortalidone and Hydrochlorothiazide in Prevention of Cardiovascular Diseases

**DOI:** 10.31083/j.rcm2510380

**Published:** 2024-10-24

**Authors:** Xiang-Ning Song, Liang Wang, Zhu-Jun Shen

**Affiliations:** ^1^Department of Cardiovascular Medicine, Peking Union Medical College Hospital, 100730 Beijing, China

**Keywords:** chlortalidone, hydrochlorothiazide, cardiovascular diseases

## Abstract

**Background:**

The variance between guideline recommendations and real-world usage might stem from the perception that chlorthalidone poses a higher risk of adverse effects, although there is no clear evidence of disparities in cardiovascular outcomes. It is crucial to assess both the clinical cardiovascular effects and adverse reactions of both drugs for clinical guidance. In this study, we present a comprehensive and updated analysis comparing the efficacy and safety of chlorthalidone (CHLOR) versus hydrochlorothiazide (HCTZ) for the prevention of cardiovascular diseases through lower the blood pressure.

**Methods:**

We conducted a systematic literature search using reputable databases including PubMed, Embase, Cochrane, and Web of Science up to April 2023, to identify studies that compared the efficacy and safety of CHLOR versus HCTZ for the long term prognosis of cardiovascular disease. This analysis represents the most up-to-date and systematic evidence on the comparative efficacy and safety of CHLOR and HCTZ for cardiovascular diseases.

**Results:**

Our review included a total of 6 eligible articles with a cohort of 368,066 patients, of which 36,999 were treated with CHLOR and 331,067 were treated with HCTZ. The primary diagnosis studied in six articles was hypertension. Initial features between the two different groups were comparable across every possible outcome. These papers followed patients using the two drugs over a long period of time to compare the differences in the occurrence of cardiovascular disease, and the results were as follows, the confidence interval is described in square brackets, followed by the *p*-value: We measured the outcomes of myocardial infarction with an odds ratio (OR) of 0.80 [0.56, 1.14], *p* = 0.41, heart failure with an OR of 0.86 [0.64, 1.14], *p* = 0.05, cardiovascular events with an OR of 1.85 [0.53, 6.44], *p* = 0.34, non-cancer-related death with an OR of 1.02 [0.56, 1.85], *p* = 0.45, death from any cause with an OR of 1.95 [0.52, 7.28], *p* = 0.32, complication rate, stroke with an OR of 0.94 [0.80, 1.10], *p* = 0.45, hospitalization for acute kidney injury with an OR of 1.38 [0.40, 4.78], *p* = 0.61 and hypokalemia with an OR of 2.10 [1.15, 3.84], *p* = 0.01. Pooled analyses of the data revealed that CHLOR was associated with a higher incidence of hypokalemia compared to HCTZ and the results were statistically significant.

**Conclusions:**

CHLOR and HCTZ are comparable in efficacy for prevention cardiovascular diseases, with the only difference being a higher incidence of hypokalemia in patients using CHLOR compared to those using HCTZ. Considering the potential heterogeneity and bias in the analytical studies, these results should be interpreted with caution.

## 1. Introduction

Hypertension impacts an estimated 116 million adults in the U.S. and over a billion adults globally, making it a primary cause of morbidity and mortality related to cardiovascular disease [1]. Chlorthalidone (CHLOR) has been shown to have a good anti hypertensive effect in several prospective studies because of its long half-life, especially in combination with multiple antihypertensive drugs, and is considered to be superior to hydrochlorothiazide (HCTZ) as an antihypertensive agent [2]. CHLOR is 1.5 to 2 times as effective as HCTZ in lowering blood pressure (BP) at equivalent doses, and has better cardiovascular outcomes [3]. However, frequent use of CHLOR can also aggravate electrolyte disorders, such as hypokalemia and hyponatremia, which may be difficult to correct and curtails its use [4]. The key mechanism by which thiazide diuretics achieve their ability to lower BP is by inhibiting the electroneutral sodium chloride transporter present in the apical membrane of the initial segment of the distal tubule. By impeding the re-uptake of sodium at this location, the delivery of sodium to the collecting duct is enhanced, which increases natriuresis as well as the exchange with potassium and magnesium [5]. Evidence from a large-scale study suggests that CHLOR is not only associated with hypokalemia but may also increase the risk of acute and chronic kidney failure and diabetes [6]. Given the crucial role of prompt BP lowering and mitigating adverse side effects in determining a suitable choice of therapy for hypertension management, it is vital to conduct a rigorous analysis comparing the efficacy and safety of the two medications in an unbiased fashion [7]. This study aims to assess the efficacy and safety of CHLOR and HCTZ for the prevention of cardiovascular diseases using systematic review and meta-analysis methodology, to provide evidence-based guidance for the use of these two antihypertensive agents in clinical practice.

## 2. Methods

### 2.1 Search Strategy and Selection

This evidence-driven analysis was performed using the PRISMA 2020 guidelines (Preferred Reporting Items for Systematic Reviews and Meta-Analysis) [8] (**Supplementary Table 1**) and was prospectively registered in the PROSPERO (CRD42023416670, https://www.crd.york.ac.uk/PROSPERO/display_record.php?RecordID=416670). We conducted the search using data from PubMed, Cochrane, Web of Science and Embase, up to April 2023. The included English language studies compared the efficacy and safety of CHLOR and HCTZ for the treatment of cardiovascular diseases. We reviewed databases using the following terms: “Chlorthalidone”, “Chlorphthalidolone”, “Phthalamudine”, “Oxodoline”, “Chlortalidone”, “Hygroton”, “Thalitone”, “Hydrochlorothiazide”, “HCTZ”, “Dichlothiazide”, “Dihydrochlorothiazide”, “HydroDIURIL”, “Oretic”, “Sectrazide”, “Esidrix”, “Esidrex”, “Hypothiazide”, “cardiovascular diseases”, “Cardiovascular Disease”, “Disease, Cardiovascular”, “Major Adverse Cardiac Events”, “Cardiac Events”, “Cardiac Event”, “Event, Cardiac”, “Adverse Cardiac Event”, “Adverse Cardiac Events”, “Cardiac Event, Adverse” and “Cardiac Events, Adverse ”. The comprehensive search strategy is shown in **Supplementary Table 2**. To ensure the completeness of our literature search, the lists of all eligible references were also examined manually. Both investigators systematically and independently evaluated each included study. Any discrepancies in the search process were addressed by mutual discussion and consent.

### 2.2 Inclusion and Exclusion Criteria

Inclusion criteria were defined as follows: (1) the study design must have been randomized, cohort or case-control; (2) the study enrolled adult participants with diagnosed cardiovascular diseases; (3) the study compared the effectiveness of CHLOR and HCTZ; (4) the study must have reported at least one of the following clinical outcomes: myocardial infarction (MI), heart failure (HF), non-cancer-related death, stroke, death from any cause, hypokalemia, hospitalization for acute kidney injury, or cardiovascular events; and (5) sufficient data was available to allow calculation of a weighted mean difference (WMD) or an odds ratio (OR). This study eliminated non-original articles such as editorial comments, letters, case reports, reviews, pediatric articles, and conference abstracts. Additionally, only published articles were included. Articles written in languages other than English were not considered for inclusion.

### 2.3 Data Extraction

All data collection was carried out independently by two investigators, with any discrepancies resolved through discussion and final decisions made by a third investigator. The following data were extracted from the included article: publication year, first author, study period, original country, design of study, size of sample, participant demographics including age, gender and body mass index (BMI), as well as specific clinical outcomes such as glomerular filtration rate (GFR) <60 mL/min/1.73 m^2^, MI, HF, non-cancer-related death, stroke, death from any cause, hypokalemia, hospitalization for acute kidney injury, and cardiovascular events. In cases where continuous variables were presented as median range or interquartile range, mean ± standard deviation was calculated using dependable mathematical methods [9, 10]. In instances where data were either lacking or not reported in the original studies, the corresponding author(s) were contacted to provide the data.

### 2.4 Quality Assessment

The quality of included articles were evaluated by The Newcastle–Ottawa Scale (NOS) [11], and those studies considered high quality received seven to nine points [12]. We assessed the quality of eligible randomized controlled trials (RCTs) based on the Cochrane Handbook for Systematic Reviews of Interventions 5.1.0, using 7 criteria. These included random sequence generation, allocation concealment, blinding, incomplete outcome data, selective reporting, and other sources of bias. Each criterion was assigned a low, high or unclear risk outcome and studies with more low-risk outcomes were considered superior [13]. Two researchers independently assessed study quality and evidence and resolved discrepancies via discussion.

### 2.5 Data Analysis

Data analysis was performed by Review Manager 5.3 (Cochrane Collaboration, Oxford, UK). WMD and OR were used for continuous and dichotomous variables. All metrics were presented with 95% CI. Study heterogeneity was assessed using χ^2^ test (Cochran’s Q) and I^2^ index [14]. Significant heterogeneity was determined by either χ^2^, *p* value < 0.05 or I^2^ >50%. A random-effects model was applied for combined WMD or OR estimations in cases where assignable heterogeneity was detected. Conversely, the fixed effect model was used for estimations when heterogeneity was not significant. To evaluate the effect of the studies with significant heterogeneity, one-way sensitivity analyses were performed. We evaluated publication bias visually using funnel plots in Review Manager 5.3 version (Cochrane Collaboration, Oxford, UK) and Egger’s regression tests were conducted using Stata 12.0 version (Stata Corp, College Station, TX, USA) for results that have been studied in 3 or more trials. A *p* value < 0.05 for publication bias was considered statistically significant.

## 3. Results

### 3.1 Characteristic

**Fig. 1.**
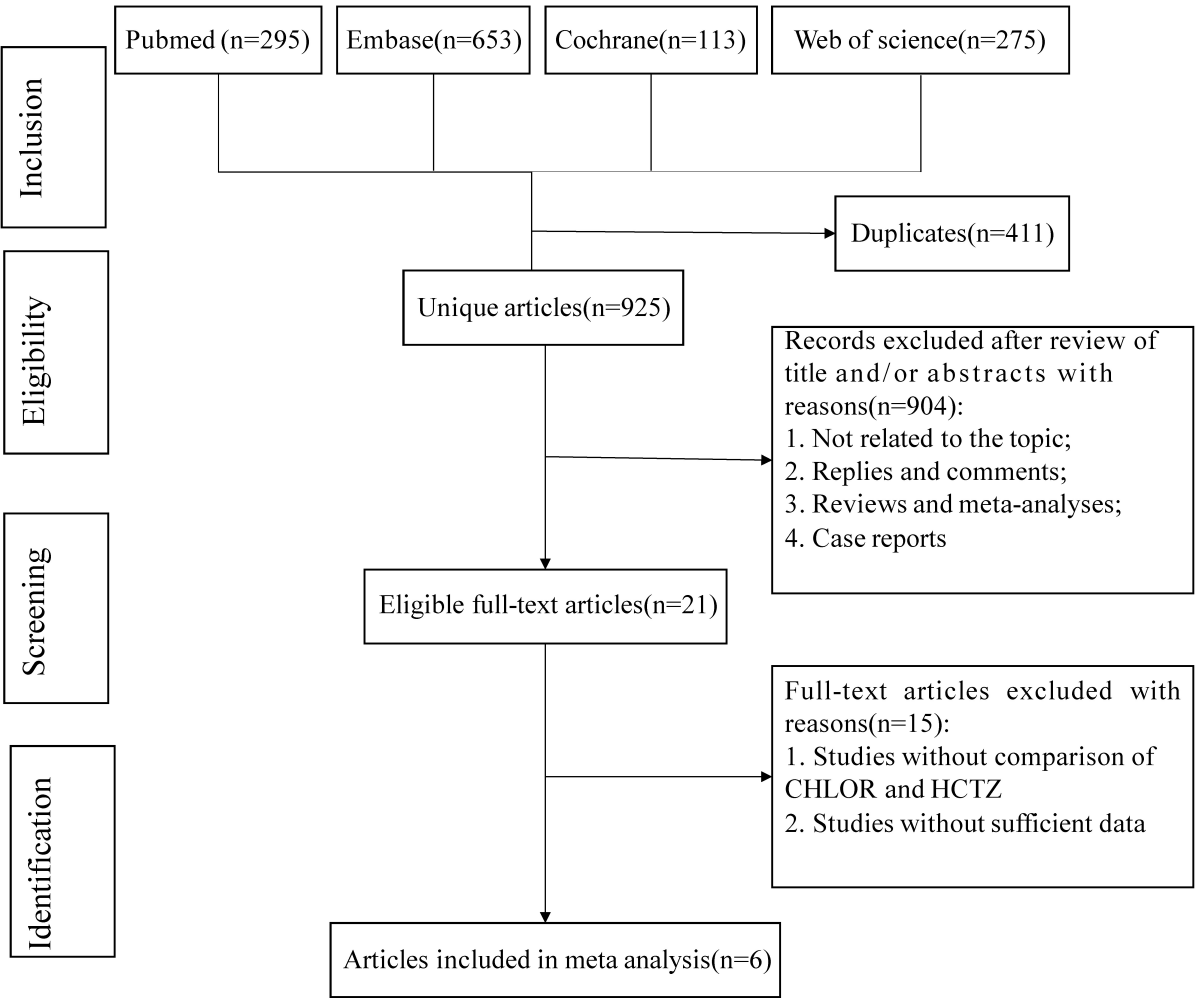
**Flowchart of the systematic search and selection process**. HCTZ, hydrochlorothiazide; CHLOR, chlorthalidone.

The search and selection process is summarized in Fig. 1. In total, 1336 articles were identified from PubMed (n = 295), Embase (n = 653), Cochrane (n = 113), and Web of Science (n = 275). After removing duplicates, 925 titles and abstracts were screened, leading to 6 full-text articles involving 368,066 patients (36,999 CHLOR vs. 331,067 HCTZ) for pooled analysis. Among them, 5 were retrospective cohort studies, and 1 was a prospective, randomized study. Table 1 (Ref. [2, 15, 16, 17, 18, 19]) provides the characteristics and quality score of each study; 5 were identified as good quality, with a median (range) score of 7. **Supplementary Figs. 1,2** demonstrate the risk of bias for 1 RCT study. **Supplementary Table 3 **presents details of the quality assessment for all the eligible studies.

**Table 1.** **Baseline characteristics of include articles and methodological**.

Authors	Study period	Country	Study design	Median follow-up (months)	Quality score	Primary diagnosis	Groups	Dose	Patients (n)	Female (%)	Age	Abnormal kidney function (%)	Diabetes (%)	Other antihypertensive drugs
Ishani *et al*. [2] 2022	2016–2021	USA	RCT	28	/	Hypertension	CHLOR	12.5 mg/25 mg	6756	6536 M (96.7)	72.4 ± 5.4	1550 (22.9)	2967 (43.9)	ACE inhibitors, ARBs, Calcium channel blockers, β-Blockers
							HCTZ	25 mg/50 mg	6767	6556 M (96.9)	72.5 ± 5.3	1547 (22.9)	3062 (45.2)	
Edwards *et al*. [18] 2021	2007–2015	Canada	Retrospective	12	8	Hypertension	CHLOR	12.5 mg/25 mg/50 mg	2936	1599 (54)	74 (7)	914 (31)	1322 (45)	ACE inhibitors, ARBs, Calcium channel blockers, β-Blockers
							HCTZ	12.5 mg/25 mg/50 mg	9786	5464 (56)	74 (7)	2131 (26)	4122 (42)	
Dhalla *et al*. [15] 2013	1993–2010	Canada	Retrospective	21	8	Hypertension	CHLOR	12.5 mg/25 mg/50 mg	10,384	6160 (59.3)	73 (6.0)	42 (0.2)	22 (0.2)	ACE inhibitors, ARBs, Calcium channel blockers, β-Blockers
							HCTZ	12.5 mg/25 mg/50 mg	19,489	11,505 (59.0)	73 (5.9)	2691 (14)	42 (0.2)	
Dorsch *et al*. [19] 2011	1973–2010	USA	Retrospective	72	8	Hypertension	CHLOR	>50 mg/≤50 mg	2392	0	46.7 ± 5.7	/	1563 (65.3)	Antiadrenergic drug therapy, an arteriolar vasodilator, and then guanethidine
							HCTZ	>50 mg/≤50 mg	4049	0	46.9 ± 5.9	/	2740 (67.7)	
Hripcsak *et al*. [16] 2020	2001–2018	USA	Retrospective	/	8	Hypertension	CHLOR	12.5 mg/25 mg	14,317	7310 (51.8)	49.0 (10.4)	140 (1.0)	630 (4.5)	ACE inhibitors, ARBs
							HCTZ	12.5 mg/25 mg	290,334	175,600 (61.1)	48.2 (10.6)	1400 (0.5)	13,200 (4.6)	
Saseen *et al*. [17] 2015	2005–2012	USA	Retrospective	12	6	Hypertension	CHLOR	25 mg/25 mg	214	53.3	58.8	0.9	14.5	ACE inhibitors, ARBs, Calcium channel blockers, β-Blockers
							HCTZ	25 mg/50 mg	642	54.7	59.1	0.9	14	

CHLOR, chlorthalidone; RCT, randomized controlled trial; HCTZ, hydrochlorothiazide; M, male; ACE, angiotensin converting enzyme inhibitor; ARBs, angiotensin receptor blockers.

### 3.2 Demographic Characteristics

The groups did not differ significantly in age (WMD 0.08; 95% CI–0.01, 0.17; *p* = 0.07), gender (male/total, OR 1.00; 95% CI 0.98, 1.03; *p* = 0.80), or BMI (WMD –0.04; 95% CI –0.17, 0.09; *p* = 0.51). However, they differed significantly in baseline GFR <60 mL/min/1.73 m^2^ (WMD 1.15; 95% CI 0.88, 1.51; *p* = 0.30) (Table 2).

**Table 2.** **Clinical characteristics and demographics of included articles**.

Items	Articles	No. of patients	WMD or OR	95% CI	*p*-value	Heterogeneity			
		CHLOR/HCTZ				Chi^2^	df	*p*-value	*I*^2^ (%)
Age (years)	5	36,785/360,425	0.08	[–0.01, 0.17]	0.07	31.09	4	<0.00001	87
Gender (male)	6	36,999/331,067	1.00	[0.98, 1.03]	0.80	6.28	4	0.18	36
BMI (kg/m^2^)	2	9148/10,816	–0.04	[–0.17, 0.09]	0.51	0.56	1	0.46	0
GFR <60 mL/min/1.73 m^2^	2	9692/16,553	1.15	[0.88, 1.51]	0.30	20.02	1	<0.00001	95

Statistically significant. BMI, body mass index; eGFR, estimated glomerular filtration rate; OR, odds ratio; CI, confidence interval; WMD, weighted mean difference; CHLOR, chlorthalidone; HCTZ, hydrochlorothiazide.

### 3.3 Myocardial Infarction (MI)

**Fig. 2.**
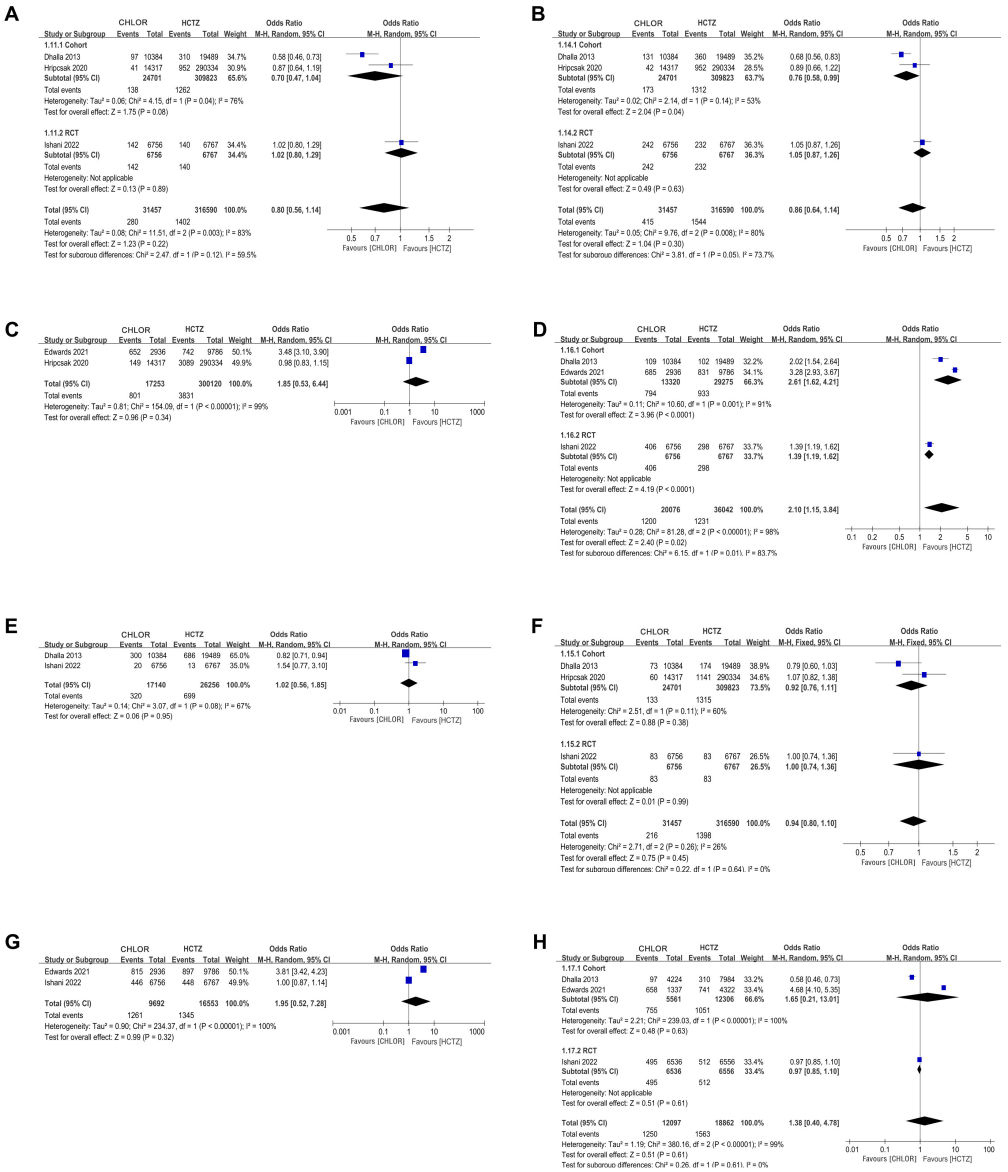
**Forest plots of perioperative outcomes**. (A) Myocardial infarction. (B) Heart failure. (C) Cardiovascular event. (D) Hypokalemia. (E) Non-cancer-related death. (F) Stroke. (G) Death of any cause. (H) Hospitalization for acute kidney injury. CHLOR, chlorthalidone; HCTZ, hydrochlorothiazide; RCT, randomized controlled trial; M-H, Mantel-hanszel.

**Fig. 3.**
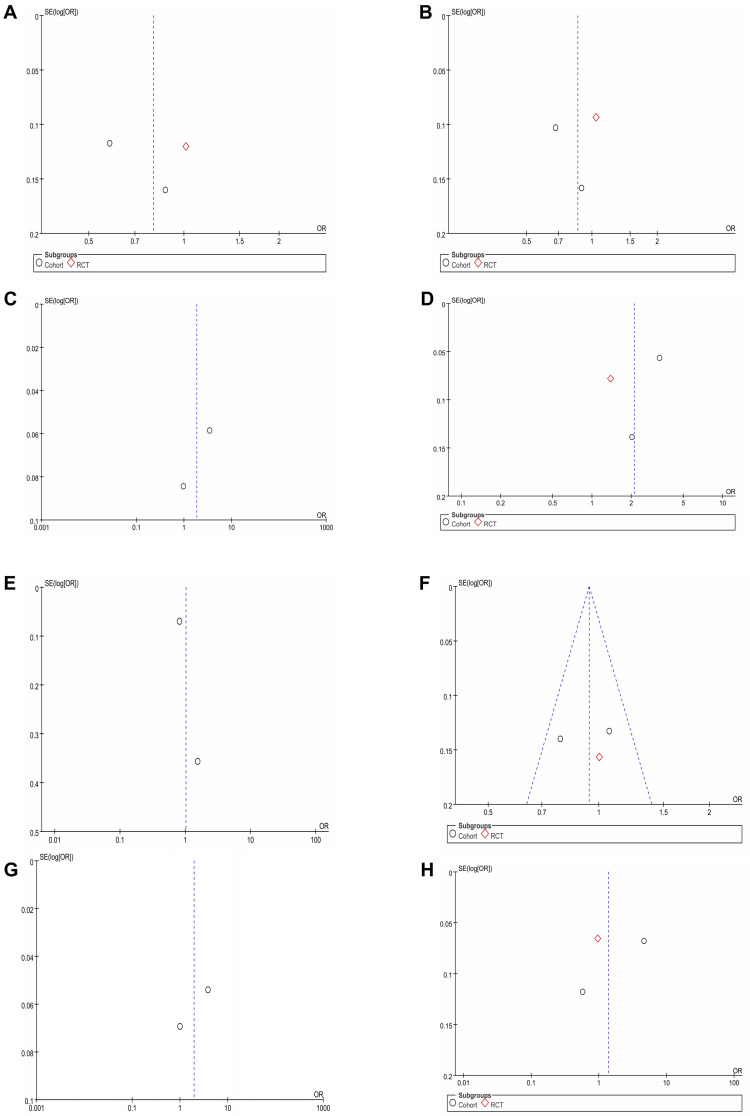
**Funnel plots of perioperative outcomes**. Funnel plots of (A) Myocardial infarction. (B) Heart failure. (C) Cardiovascular event. (D) Hypokalemia. (E) Non-cancer-related death. (F) Stroke. (G) Death of any cause. (H) Hospitalization for acute kidney injury. RCT, randomized controlled trial.

**Fig. 4.**
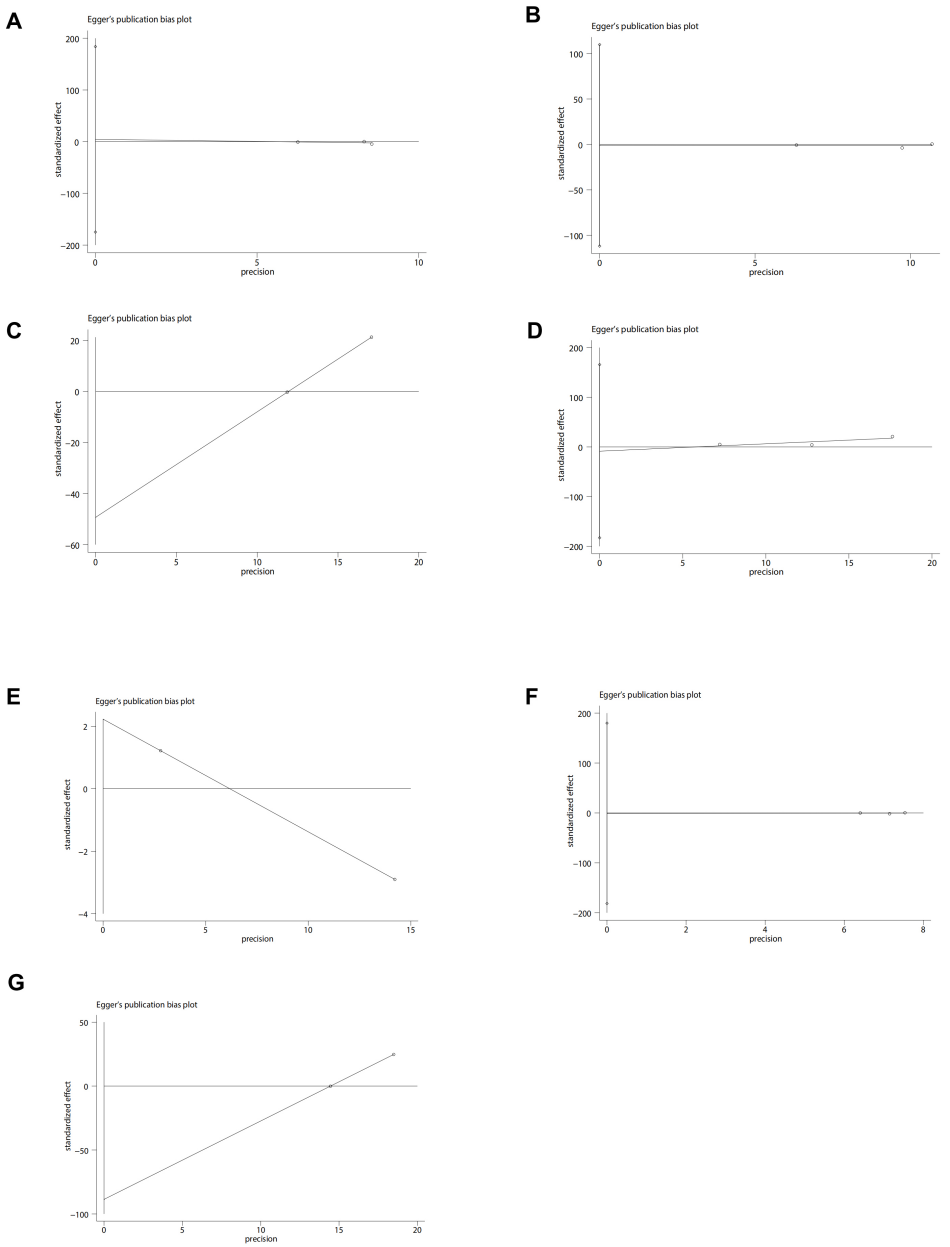
**Egger plots of perioperative outcomes**. (A) Myocardial infarction. (B) Heart failure. (C) Cardiovascular event. (D) Hypokalemia. (E) Non-cancer-related death. (F) Stroke. (G) Death of any cause.

MI data were reported in 3 studies [2, 15, 16] for a total of 348,047 patients (31,457 CHLOR vs. 316,590 HCTZ). Pooled results showed that there was no significant difference between the two groups (OR 0.80; 95% CI 0.56, 1.14; *p* = 0.41), yet there was significant heterogeneity (I^2^ = 83%, *p* = 0.03) (Fig. 2A). No visual (Fig. 3A) or statistical (Egger’s test, *p* = 0.802, Fig. 4A) publication bias was found. The exclusion of Dhalla 2013 data eliminated heterogeneity associated with MI (I^2^ = 0%, *p* = 0.45), indicating that this article was the primary factor contributing to heterogeneity in the analysis.

### 3.4 Heart Failure (HF)

The analysis included three studies [2, 15, 16] comprising 348,047 patients (31,457 CHLOR vs. 316,590 HCTZ), revealing a similar occurrence of HF events between the two groups (OR 0.86; 95% CI 0.64, 1.14; *p* = 0.05) (Fig. 2B), but with substantial heterogeneity (I^2^ = 80%, *p* = 0.008). No visual (Fig. 3B) or statistical evidence of publication bias was found (Egger’s test, *p* = 0.93, Fig. 4B). Eliminating Dhalla 2013 data removed heterogeneity associated with heart failure (I^2^ = 0%, *p* = 0.39), indicating that this study was the primary contributor to heterogeneity in the analysis.

### 3.5 Cardiovascular Events

Two articles [16, 18] reported Cardiovascular Event data in 317,373 patients (17,253 CHLOR vs. 300,120 HCTZ). There was no considerable difference in the occurrence rate between the two groups (OR 1.85; 95% CI 0.58, 6.44; *p* = 0.34) with no noticeable heterogeneity (I^2^ = 99%, *p* < 0.00001) (Fig. 2C). Funnel plots indicated a minor publication bias (Fig. 3C), conversely, the Egger’s test failed to detect any statistically significant bias (Fig. 4C).

### 3.6 Hypokalemia

Not all articles describe the definition of hypokalemia, but those that do agree that hypokalemia is defined when the blood potassium is <3.5 mmol/L. Hypokalemia analysis was performed in 3 studies [2, 15, 18] with 56,118 patients (20,076 CHLOR vs. 36,042 HCTZ), detecting significantly higher hypokalemia in the CHLOR group (OR 2.10; 95% CI 1.15, 3.84; *p* = 0.01) and heterogeneity with statistical significance (I^2^ = 97%, *p* < 0.00001) (Fig. 2D). The funnel plot showed mild publication bias (Fig. 3D), but there was no statistical significance in the Egger’s test (*p* = 0.644) (Fig. 4D).

### 3.7 Non-cancer-related Death

The analysis included two studies [2, 15] comprising 43,394 patients (17,140 CHLOR vs. 26,256 HCTZ), revealing a similar rate of non-cancer related death between the two groups (OR 1.02; 95% CI 0.56, 1.85; *p* = 0.45), and meaningful heterogeneity (I^2^ = 68%, *p* = 0.08) (Fig. 2E). Both the funnel plot (Fig. 3E) and Egger’s test (Fig. 4E) detected publication bias.

### 3.8 Stroke

Stroke data were reported in three articles [2, 15, 16] involving 348,047 patients (31,457 CHLOR vs. 316,590 HCTZ), and no marked difference was demonstrated within the groups (OR 0.94; 95% CI 0.80, 1.10; *p* = 0.45), and no meaningful heterogeneity (I^2^ = 26%, *p* = 0.26) (Fig. 2F). Funnel plots showed no publication bias (Fig. 3F), and no statistical significance was observed in the Egger’s test (*p* = 0.96) (Fig. 4F).

### 3.9 Death from Any Cause

Data of death from any cause were available in 2 studies [2, 18] totaling 26,245 patients (9692 CHLOR vs. 16,553 HCTZ). Pooled analysis revealed no significant difference in the CHLOR group in comparison to the HCTZ group (OR 1.95; 95% CI 0.52, 7.28; *p* = 0.32) with significant heterogeneity (I^2^ = 100%, *p* < 0.00001) (Fig. 2G). No visual (Fig. 3G) or statistical (Fig. 4G) evidence of publication bias was detected.

### 3.10 Hospitalization for Acute Kidney Injury

Three studies [2, 15, 18] involving 30,959 patients (12,097 CHLOR vs. 18,862 HCTZ) were included in the analysis. Pooled results demonstrated that the rate of hospitalization for acute kidney injury showed that the two groups had similar results (OR 1.38; 95% CI 0.40, 4.78; *p* = 0. 61), and significant heterogeneity was observed (I^2^ = 99%, *p* < 0.00001) (Fig. 2H). Visual (Fig. 3H) or statistical (Egger’s test), evidence of publication bias were detected. Upon excluding the data reported by Edwards 2021, the heterogeneity observed with regards to hospitalization for acute kidney injury was reduced (I^2^ = 93%, *p* < 0.00001), suggesting that this study was the principal origin of heterogeneity in the analysis.

### 3.11 Sensitivity Analyses

**Fig. 5.**
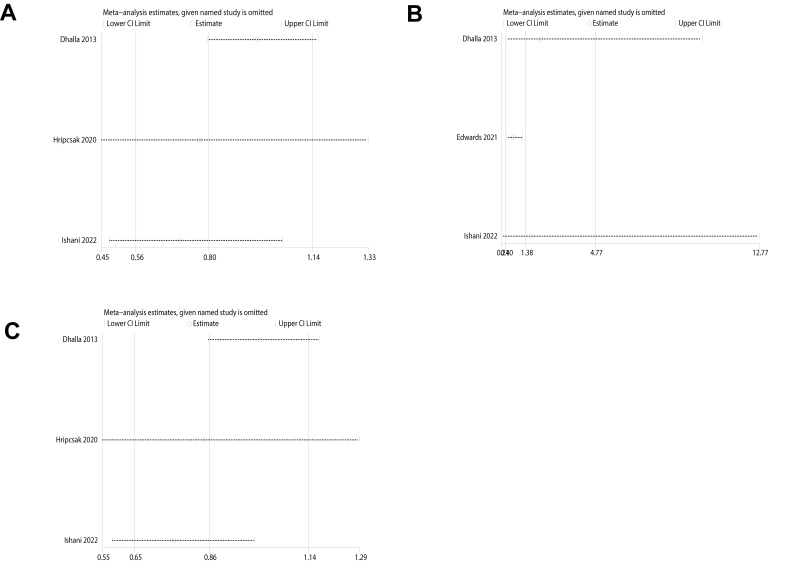
**Sensitivity analysis**. Sensitivity analysis of (A) Myocardial infarction, (B) Heart failure, (C) Hospitalization for acute kidney injury.

This study conducted a sensitivity analysis using hospitalization rates for MI, HF, and acute kidney injury, which displayed considerable heterogeneity. By systematically excluding studies, it was found that the heterogeneity for MI (Fig. 5A) and acute kidney injury hospitalization rates (Fig. 5B) did not significantly decrease, indicating stability in the results. However, upon the exclusion of the paper by Dhalla IA, there was a significant reduction in heterogeneity (I^2^ = 0%, *p* = 0.39), demonstrating that this particular study was significantly heterogeneous compared to other studies regarding the HF endpoint (Fig. 5C). Nevertheless, the meta-analysis results for the HF endpoint remained consistent, with no statistical difference before and after the exclusion of this study.

## 4. Discussion

Cardiovascular disease affects a substantial proportion of the global population, including coronary, cerebrovascular, and renal diseases. Reducing elevated BP levels has been shown to lessen the chance of cardiovascular and renal complications in patients with hypertension [20]. Thiazines are widely used because of their good antihypertensive effect [21] the first-line antihypertensive agent are effective in lowering blood pressure and reducing adverse cardiovascular outcomes [22]. Most clinical trials have focused on the dose and antihypertensive effects of these two drugs, but have not consistently followed patients for long-term outcomes [23]. Because the use of diuretic drugs in cardiovascular disease guidelines is based on single factors, this article provides advice for clinical decision-making and medication, focusing on the long-term effects of the two most commonly used thiazide drugs on the cardiovascular system [24]. A detailed comparison was made between the two drugs with the same antihypertensive effect and whether there was a statistically significant difference in future death from MI, HF, cerebral infarction, kidney injury or other non-tumor factors [25]. In addition to efficacy, safety is also a key concern. It has been recognized that thiazide diuretics can cause hypokalemia and hyponatremia to different degrees [26]. In articles discussing related issues, there was a significant increase in hospitalization rates for hypokalemia and hyponatremia in patients receiving CHLOR, with those patients being 3 times more likely to be hospitalized with hypokalemia and approximately 1.7 times more likely to be hospitalized with hyponatremia than those prescribed HCTZ [15]. The LEGEND study found no significant difference in the risk of MI, hospitalized HF, or stroke in patients treated with CHLOR vs. HCTZ [16]. However, CHLOR was associated with significantly higher risks of hypokalemia [23]. In another large study with 29,873 participants, CHLOR did not significantly reduce the composite outcome of death, hospitalization for HF, stroke, or MI compared to HCTZ, with an increased incidence of hypokalemia and hyponatremia [15]. The statistical results in this paper support the above conclusion, and HCTZ appears to be safer and just as effective. We came across similar articles in our research, but they did not discuss in detail the OR value, heterogeneity, or sensitivity analyses of the occurrence of individual events.

Despite these important findings, our study has certain limitations. First, the number of studies included was small and was comprised primarily of cohort studies with only one RCT. To ensure reliability, this research accessed four English-language databases; unfortunately, only six publications met our inclusion criteria [17, 18, 22]. Second, while sensitivity investigations were executed to evaluate the stability of the results, potential confounding factors could still affect the outcomes. These include but are not limited to baseline characteristics of the patients, comorbidities, and specific interventions. Third, some outcome measures, such as cardiovascular events and hypokalemia, exhibited slight publication bias in funnel plots, although the Egger’s test did not indicate statistically significant publication bias.

Given these limitations, future research should include more RCTs to provide stronger evidence supporting the comparison between the two drugs. Moreover, future study designs should be more rigorous, to minimize potential biases and confounders, thereby enhancing the reliability and general applicability of the research findings.

## 5. Conclusions

The summary analysis shows that effects of CHLOR and HCTZ on MI, HF, cardiovascular events, non-cancer-related death, death from any cause, stroke, and hospitalization for acute kidney injury were not significantly different, but CHLOR had a higher risk of hypokalemia. Given the manifestation of heterogeneity and potential bias, physicians should choose the appropriate blood pressure medication based on their experience and individual patient factors.

## Availability of Data and Materials

Data sharing is not applicable as no data were generated or analyzed.

## Supplementary Material

Supplementary material associated with this article can be found, in the online version, at https://doi.org/10.31083/j.rcm2510380.
