# Supplementary material for: Efficacy and Safety of Chlortalidone and Hydrochlorothiazide in Prevention of Cardiovascular Diseases
Source: Rev Cardiovasc Med. 2024 Oct 24;25(10):380. doi: 10.31083/j.rcm2510380 (PMC11522762; doi:10.31083/j.rcm2510380)
Supplement: Supplementary file 1 [file 2153-8174-25-10-380-s1.zip › Supplementary Fig. 2.pdf]

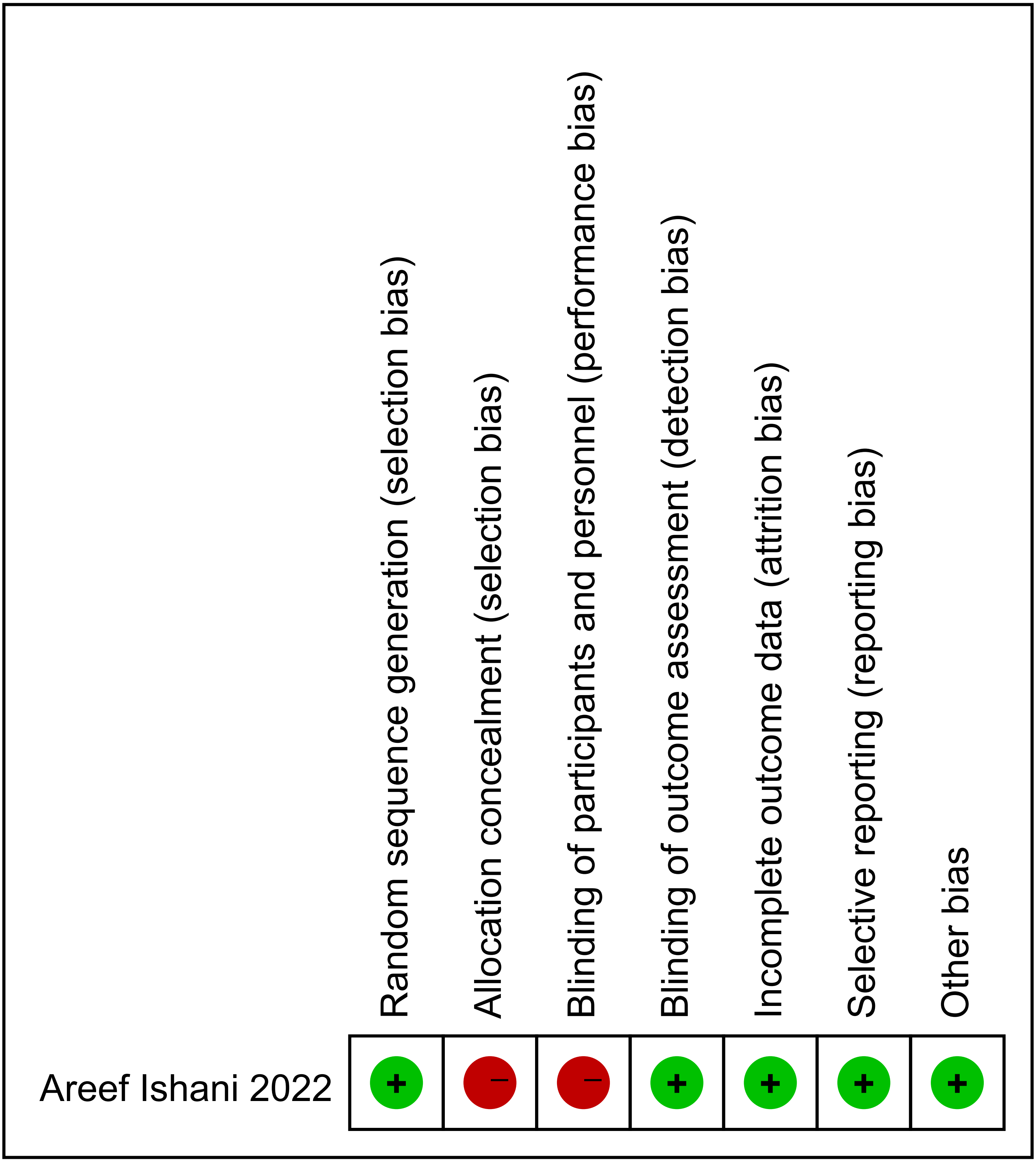
Random sequence generation (selection bias)
Allocation concealment (selection bias)
Blinding of participants and personnel (performance bias)
Blinding of outcome assessment (detection bias)
Incomplete outcome data (attrition bias)
Selective reporting (reporting bias)
Other bias
Areef Ishani 2022
+
–
–
+
+
+
+
