# Supplementary material for: Efficacy and Safety of Chlortalidone and Hydrochlorothiazide in Prevention of Cardiovascular Diseases
Source: Rev Cardiovasc Med. 2024 Oct 24;25(10):380. doi: 10.31083/j.rcm2510380 (PMC11522762; doi:10.31083/j.rcm2510380)
Supplement: Supplementary file 1 [file 2153-8174-25-10-380-s1.zip › Supplementary Table 2.pdf]

Supplementary Table S2. Detailed search strategy in three databases.

Database	Search strategy
Pubmed	((((((((Chlorphthalidolone) OR (Phthalamudine)) OR (Oxodoline)) OR (Chlortalidone)) OR (Hygroton)) OR (Thalitone)) OR ("Chlorthalidone"[Mesh])) AND ((((((((((HCTZ) OR (Dichlothiazide)) OR (Dihydrochlorothiazide)) OR (HydroDIURIL)) OR (Oretic)) OR (Sectrazide)) OR (Esidrix)) OR (Esidrex)) OR (Hypothiazide)) OR ("Hydrochlorothiazide"[Mesh]))) AND (((((((((((Cardiovascular Disease) OR (Disease, Cardiovascular)) OR (Major Adverse Cardiac Events)) OR (Cardiac Events)) OR (Cardiac Event)) OR (Event, Cardiac)) OR (Adverse Cardiac Event)) OR (Adverse Cardiac Events)) OR (Cardiac Event, Adverse)) OR (Cardiac Events, Adverse)) OR (Cardiovascular Diseases))
Cochrane	1 (Chlorthalidone or Chlorphthalldolane or Phthalamudine or Oxodoline or Chlortalidone or Hygroton ar Thalitone).af.<br>2 (hydrochlorothiazide ar HcT2 or Dichlothiazide ar Dlhydrochlorothiazide or HydraDluRi. or Orellc: ar Sectrazide ar Esidix orEsidrex or Hypothiazide).af.<br>3(cardovascular diseases or Cardlovascular Disease or Disease, Cardkovascular or Malor Adverse Cardlac Events or Cardlac Eventsor Cardlac Event or Event, Cardlac or Adverse Cardlac Event or Advese Cardiac Events or Cardlac Event, Adverse or Cardlac Events.Adverse).af.<br>1 and 2 and 3
Embase*	1 (Chlorthalidone or Chlorphthalldolane or Phthalamudine or Oxodoline or Chlortalidone or Hygroton ar Thalitone).af.<br>2 (hydrochlorothiazide ar HcT2 or Dichlothiazide ar Dlhydrochlorothiazide or HydraDluRi. or Orellc: ar Sectrazide ar Esidix orEsidrex or Hypothiazide).af.<br>3(cardovascular diseases or Cardlovascular Disease or Disease, Cardkovascular or Malor Adverse Cardlac Events or Cardlac Eventsor Cardlac Event or Event, Cardlac or Adverse Cardlac Event or Advese Cardiac Events or Cardlac Event, Adverse or Cardlac Events.Adverse).af.<br>1 and 2 and 3
Web of Science	Web of science<br>1 Chlorthalidone (Topic)or Chlorphthalldolane (Topic)or Phthalamudine (Topic)or Oxodoline (Topic)or Chlortalidone (Topic)or Hygroton ar Thalitone<br>2 hydrochlorothiazide ar HcT2 (Topic)or Dichlothiazide ar Dlhydrochlorothiazide (Topic)or HydraDluRi. (Topic)or

		Orellc: ar Sectrazide ar Esidix orEsidrex or Hypothiazide
		3 cardovascular diseases (Topic)or Cardlovascular Disease (Topic)or Disease, Cardkovascular (Topic)or Malor Adverse Cardlac Events (Topic)or Cardlac Events(Topic)or Cardlac Event (Topic)or Event, Cardlac (Topic)or Adverse Cardlac Event (Topic)or Advese Cardiac Events (Topic)or Cardlac Event, Adverse (Topic)or Cardlac Events.Adverse
		((#1)and 2) and# 3

* We retrieved articles from Embase via the Ovid (https://ovidsp.ovid.com/).
