# Supplementary material for: Efficacy and Safety of Chlortalidone and Hydrochlorothiazide in Prevention of Cardiovascular Diseases
Source: Rev Cardiovasc Med. 2024 Oct 24;25(10):380. doi: 10.31083/j.rcm2510380 (PMC11522762; doi:10.31083/j.rcm2510380)
Supplement: Supplementary file 1 [file 2153-8174-25-10-380-s1.zip › Supplementary Table 3.pdf]

Supplementary Table S3. Quality evaluation of the eligible studies with Newcastle–Ottawa scale.

Study	Selection				Comparability		Outcome		
	Representative-ness	Selection of non-exposed	Ascertainment of exposure	Outcome not present at start	Comparability on most important factors	Comparability on other risk factors	Assessment of outcome	Long enough follow-up (median≥1 year)	Adequacy (completeness) of follow-up
Saseen 2015	-	*	*	-	*	-	*	*	*
Edwards 2021	*	*	*	-	*	*	*	*	*
Dhalla 2013	*	*	*	-	*	*	*	*	*
Dorsch 2011	*	*	*	-	*	*	*	*	*
Hripcsak 2020	*	*	*	-	*	*	*	*	*

*indicates criterion met; - indicates significant of criterion not met.
