# Supplementary figures and images for: Efficacy and Safety of Chlortalidone and Hydrochlorothiazide in Prevention of Cardiovascular Diseases
Source: Rev Cardiovasc Med. 2024 Oct 24;25(10):380. doi: 10.31083/j.rcm2510380 (PMC11522762; doi:10.31083/j.rcm2510380)

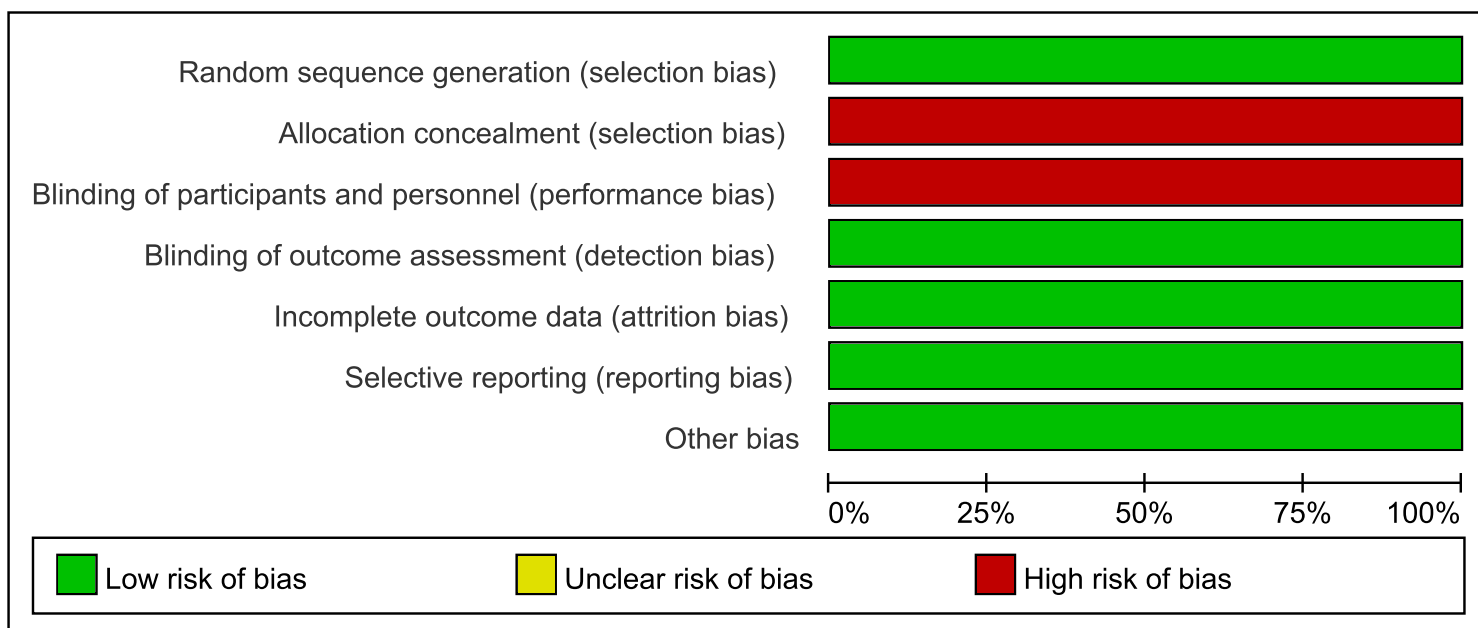

Supplement: Supplementary file 1 [file 2153-8174-25-10-380-s1.zip › Supplementary Fig. 1.pdf]
